# Bluebelle study (phase A): a mixed-methods feasibility study to inform an RCT of surgical wound dressing strategies

**DOI:** 10.1136/bmjopen-2016-012635

**Published:** 2016-09-22

**Authors:** The Bluebelle Study Group, the Severn and Peninsula Audit and Research Collaborative for Surgeons, and the West Midlands Research Collaborative

**Affiliations:** School of Social and Community Medicine, University of Bristol, Bristol, UK

**Keywords:** QUALITATIVE RESEARCH, SURGERY, Feasibility Studies, WOUND MANAGEMENT

## Abstract

**Objectives:**

Dressing primary surgical wounds is common, but the implications for surgical site infection (SSI) remain unknown. The Bluebelle study aimed to determine the feasibility of a randomised controlled trial (RCT) comparing ‘simple’, ‘complex’ or ‘no’ dressings on abdominal wounds, as prespecified in a funder's research brief. Bluebelle includes exploratory work (phase A) to inform a pilot version of the proposed RCT (phase B). Phase A aimed to investigate current dressing practices and perspectives on the proposed RCT, with a view to refining the forthcoming pilot.

**Design:**

Mixed methods, including semi-structured interviews and document analysis.

**Setting:**

6 UK hospitals.

**Participants:**

51 patients and 92 clinical professionals from abdominal surgical specialities.

**Results:**

Professionals had variable interpretations of what constitutes a ‘dressing’, particularly with respect to ‘glue’—a product listed under ‘wound-closure products’ in the British National Formulary, which some surgeons reportedly applied as a ‘wound covering’. Areas of ambiguity arising from interviews informed development of pragmatic definitions, including specification of conditions under which glue constituted a ‘dressing’. Professionals reported that ‘simple’ dressings were routinely used in practice, whereas ‘complex’ dressings were not. This raised questions about the relevance of comparison groups, prompting the design of a survey to determine the types/frequency of dressing use in abdominal surgery (reported elsewhere). This confirmed that complex dressings were rarely used, while ‘glue as a dressing’ was used relatively frequently. ‘Complex dressings’ were therefore substituted for ‘glue as a dressing’ (following an updated Cochrane review, which found insufficient evidence to determine the effectiveness of ‘glue as a dressing’). Patients and professionals acknowledged uncertainty around dressing use and SSI prevention, but felt dressings may serve practical and/or psychological benefits. This steered development of additional outcome measures for the pilot.

**Conclusions:**

Pre-trial qualitative research can highlight areas of ambiguity and inform new lines of enquiry in relation to prespecified research briefs, enabling adjustments to RCT design that enhance relevance to practice.

**Strengths and limitations of this study**

- The study presents a comprehensive and practical account of the ways in which qualitative research instigated key changes to the design of a prespecified randomised controlled trial (RCT) of surgical wound dressing strategies.
- The reporting of the research process is novel, emphasising the fluid and flexible nature of investigations that were necessary in light of emerging questions surrounding the relevance of the funder's research brief.
- The clinical context of the study enabled detailed exploration of the reasons underpinning postsurgical wound dressing practices—an area that was previously poorly understood.
- Patients' and clinicians' reported acceptability of an RCT of wound dressing strategies were based on hypothetical contexts, thus limiting the potential to understand actual reactions to a future RCT.

## Introduction

Millions of operations are performed annually worldwide. Surgical procedures often culminate in the wound edges being brought together and secured using stitches, clips, staples or tissue adhesive (‘glue’). Applying a dressing over a surgically closed (primary) wound is common after many operative procedures, although there is no evidence-based rationale for this practice.^1^ A recent Cochrane review concluded that there was insufficient evidence to recommend whether it is better to apply a dressing over primary wounds or leave these exposed to air (ie, without a dressing).^2^ The review also found no evidence to recommend any particular type of dressing over others. The studies collated in the review considered a range of primary and secondary outcomes, including appearance of scarring, pain control, patient acceptability and surgical site infection (SSI) rates.

SSI prevention is a particular priority for healthcare providers and policymakers due to the considerable burden wound infections can inflict on patients and healthcare systems.^3–5^ As such, one of the recommendations of the Cochrane review was for future randomised controlled trials (RCTs) to investigate the impact of dressing application on SSI prevention, focusing on surgical specialities with the highest rates of SSI (eg, gastrointestinal (GI) and obstetric procedures). The UK's National Institute for Health Research (NIHR) subsequently issued a commissioned research brief, inviting proposals for investigating the feasibility of conducting a large-scale RCT comparing ‘simple inexpensive dressings’, ‘complex costly dressings’ and ‘no dressings’ on surgical wounds (see online supplementary information 1). The Bluebelle feasibility study (HTA 12/200/04) was funded in response to this brief. In our application for funding, we defined ‘feasibility’ as successful delivery of a pilot RCT of ‘simple’, ‘complex’ and ‘no dressings’ in the management of primary surgical wounds. A ‘pilot’ RCT in this instance was defined as a small-scale version of a larger definitive RCT, run with the intention of testing how the components of a main RCT would work in practice.^6^

10.1136/bmjopen-2016-012635.supp1 Supplementary data

The success or failure of an RCT hinges on a number of factors, including recruitment, adherence to protocols/interventions, and the relevance of the research question to key stakeholders (eg, patients, clinicians, policymakers). Surgical RCTs are notoriously difficult to deliver owing to a lack of experience in research and individual surgeon preference.^7^ Planning with these issues in mind requires a thorough understanding of the clinical setting within which a trial will be conducted. Little was known about the nature or frequency of dressing use on postoperative wounds, the rationale underpinning these practices, or patients' and professionals' perceptions of equipoise around dressing strategies.

In light of the above uncertainties, the Bluebelle study was designed to include a preparatory phase (phase A) that aimed to refine the design and protocol of the pilot RCT (phase B). Specific objectives of phase A were to: understand the reasons underpinning current choice and use of dressings; define and categorise dressings into the prespecified comparison groups, and explore patients' and professionals' views on the proposed pilot RCT. Phase A was originally intended as a qualitative study with document analysis. However, unanticipated emerging findings prompted the decision to conduct additional research, in the form of a prospective survey, and an update to the Cochrane review^1^ of dressings and SSI prevention (both reported in full elsewhere).^8^ ^9^ This paper presents the qualitative findings in full, and refers to the key findings from the additional research projects, bringing these together to report how evolving feasibility work transformed plans for the forthcoming pilot RCT.

## Methods

Phase A adopted a mixed-methods design, consisting of qualitative interviews with healthcare professionals and patients and document analysis of the British National Formulary (BNF).^10^ The BNF is a pharmaceutical reference book, available in print and online, which sets out legal and professional guidelines on prescribing medicines. Information within the BNF includes names, legal classification, recommended doses, indications/contraindications, side effects and prices of medicinal products.

The qualitative interviews and document analysis were preplanned, but findings informed further (unanticipated) data collection and lines of enquiry. This included a prospective survey (reported elsewhere)^8^ and an update to an existing Cochrane systematic review^1^ ^9^ Data from the above sources were regularly brought together during study management group and steering committee meetings to inform decisions about further investigation and the eventual design of the pilot RCT. The planned aspects of the feasibility study, and ways in which they informed further lines of enquiry and the final RCT design, are reported here.

### Qualitative interviews

Qualitative interviews investigated reasons underpinning current wound dressing practices and patients' and healthcare professionals' perspectives on the proposed pilot RCT. Fulfilment of qualitative reporting guidelines are available here (see online supplementary information 2).

10.1136/bmjopen-2016-012635.supp2 Supplementary data

#### Context

Interviews were conducted across three university-teaching National Health Service (NHS) hospitals and three district NHS hospitals in the South West and Midlands regions of England. These hospitals were selected on the basis of practical considerations (distance from the institutions hosting the research), and an intention to include a mix of hospitals (in terms of university-teaching status). There was a focus on upper/lower GI and obstetric (caesarean section) surgery, based on the Cochrane review's recommendations to focus on operations that carried a high risk of SSI,^4^ and specification of these procedures in the funder's commissioned brief. Although we did not plan to include children in the pilot RCT, interviews were also conducted in paediatric surgery (in two sites) because anecdotal information indicated that dressings were not routinely used in paediatrics. The adult versus paediatric comparison provided the potential to further the team's understanding of rationales for and against dressing use.

#### Sampling and recruitment

Eligible healthcare professionals (professionals) worked in upper/lower GI (general), obstetric and paediatric surgical specialities, or were wound care specialists. All were regularly involved in caring for surgical abdominal wounds. Eligible patients were aged over 18 years, and had undergone, or were scheduled to undergo, an abdominal surgical procedure within 3 months of the interview date. Surgical trainee research collaboratives, research nurses, and principal investigators identified eligible potential participants and sought consent to pass on contact details to the qualitative research team (LR, CM, DE, JM). As interviews progressed, sampling became increasingly purposeful to achieve maximum variation according to age, gender, type of surgery, and clinical role (professionals only). The qualitative researchers obtained written consent from patients and healthcare professionals to conduct and audio-record the interviews. Sampling and data collection ceased at the point of data saturation, defined as the point at which no new themes emerged from three consecutive interviews (assessed separately for patients and healthcare professionals).

#### Data collection

Interviews were conducted face to face or via telephone between July 2014 and July 2015 by LR, DE, CM and JM. Face-to-face interviews took place on hospital and university premises or in participants' homes. Interviews with clinical professionals lasted between 17 and 54 mins, and interviews with patients lasted between 13 and 50 mins. Variability in staff interviews was largely due to differences in clinical professionals' time constraints. Variability in patient interviews reflected differences in individuals' personal experiences of surgery, and the type of surgery they had undergone/were scheduled to undergo. These details had implications for patients' reported experiences of wound healing. Furthermore, as is often the case with qualitative research, interview participants naturally differed in the extent to which they spoke about their views and prior experiences, with some individuals offering more detail, and speaking more extensively, than others. An evolving topic guide (see online supplementary information 3) was used to ensure key topics were consistently covered, with the team regularly meeting to update its content based on emerging findings. New areas for investigation, or factors that might have influenced interview conduct or interpretation, were recorded in field notes after interviews. The interviewers regularly discussed interview content and technique during team meetings. This ensured that difficult trial concepts, such as ‘outcome measures’, were explained consistently across interviews. The team also agreed on particular strategies for building rapport with patients. For example, although interviews focused on dressing-related issues, there was agreement that researchers would begin interviews with open-ended questions about the patients' experiences of clinical care/recovery/symptoms.

10.1136/bmjopen-2016-012635.supp3 Supplementary data

#### Analysis

Interviews were transcribed and subjected to thematic analysis, guided by constant comparison methods adopted from grounded theory methodology.^11^ Transcripts were coded line by line in NVivo V.10 (QSR International Pty V.10, 2012, Victoria, Melbourne, Australia), with a subset (10%) coded by at least two researchers at the start of data collection to inform an initial coding framework. Some codes were developed *a priori*, in line with the topic guide and focused study objectives. Various levels of subcodes and additional broad codes were added inductively as analysis proceeded. Members of the qualitative team met regularly to discuss the evolving coding framework. Data were summarised in matrices to help identify patterns of responses based on participant characteristics (eg, surgical specialty, clinical role, etc). Descriptive accounts summarising emerging themes were prepared for the study management group throughout the analytical process. Negative cases (ie, participants whose views did not align with the major themes reported), were clearly identified in reports/presentations of findings.

### Document analysis

The ‘wound management products’ section of the BNF^10^ was scrutinised for relevant information about current dressing names, classifications and costs. Extracted data were summarised in tables and considered alongside data emerging from the qualitative interviews to facilitate development of pragmatic definitions for the trial comparison groups to be adopted in the forthcoming pilot RCT. Listed BNF dressings were subsequently classified into these groups.

## Results

One hundred and forty-three qualitative interviews were conducted (92 professionals, 51 patients), 102 of which focused on current wound dressings practices (69 professionals, 33 patients), and 41 on use of glue as a dressing (23 professionals, 18 patients; tables 1–3). There were no withdrawals.

**Table 1:** Role and surgical specialty of professional participants interviewed about wound dressing practices

Surgical specialty				
Participant type	General	Obstetric	Paediatric	Total
Surgeon	16	4	6	26
Registrar	6	3	3	12
Nurse/midwife	19	8	2	29
Tissue viability specialist	1	0	0	1
Community district nurse	1	0	0	1
Total	43	15	11	69

**Table 2:** Role and surgical specialty of professional participants interviewed about ‘glue as a dressing’

Surgical specialty			
Participant type	General	Obstetric	Total
Surgeon	6	3	9
Registrar	3	4	7
Nurse/midwife	0	7	7
Total	9	14	23

**Table 3:** Patient participants interviewed about ‘wound dressing practice’ and ‘glue as a dressing’ according to the site of their wounds

		Number of patients interviewed, categorised according to type of surgery received		Total
		General	Obstetric	
Focus of interview	Wound dressing practices	26	7	33
	Use of ‘glue as a dressing’	18	0	18
Total		44	7	51

### Current practice and clinical equipoise

#### Paediatrics

Paediatric professionals from both sites reported that they did not *routinely* apply dressings to primary wounds. Leaving primary wounds exposed was assumed to be common practice in paediatric surgery, although some had encountered individual paediatric consultants who did apply dressings. Most attributed their own practices to local conventions, although some rationalised their practices according to practical and psychological factors specific to the paediatric population. Children were thought to tamper with or remove dressings, experience distress on dressing removal and feel anxious at the prospect of not knowing what lies beneath a dressing:

Paediatric surgeon 1006: In paediatric surgery (we) have not been using…a lot of us have not been using dressings for ages because it's a nightmare to get them off these children again. They hate the feeling of peeling it off and having to change those every 24 hours to look at the wound and things like that. On wounds where the chances of getting infection is sort of under half a percentage or something like that, you're thinking that's probably unnecessary cruelty (laugh) in a way.

Some informants theorised that there had been no incentive or trigger to question the tradition of leaving primary wounds exposed, given the anecdotally observed low SSI rates in paediatrics. This implied an assumption that dressing application was linked with theories of SSI prevention in other specialities:

Paediatric surgeon 1008: So I think they've (children) got good cell turnover, they've got really good blood flow. They heal incredibly well, the [SSI] rates are low, so I guess there's no direct market for a company.

#### Adult surgery

In contrast to paediatrics, professionals working in adult upper/lower GI and obstetric surgery reported that dressings were routinely applied to primary wounds. Most professionals emphasised that management of primary wounds was very different to treating wounds that had been intentionally left open (‘secondary’ wounds) or wounds that had developed clinical problems. Secondary and problematic wounds were described as highly heterogeneous, with complex individual needs. Care of these wounds reportedly fell under the remit of wound specialists, who were able to make reasoned dressing selections that were tailored to the wound requirements. In contrast, dressing use in primary wounds was presented as a largely passive process that had become indoctrinated into postoperative practice, though there were isolated reports of surgeons more actively choosing a particular, comparatively novel, dressing product (‘glue’, see ‘Glue as a dressing’ section). Overall, surgeons and nurses reported little active clinical decision-making in dressing primary wounds, using whatever ‘default dressing’ had been purchased by the hospital. Although the specific dressing brands used could vary temporally and across hospitals, the default dressings described had similar characteristics: all were adhesive coverings that had no additional active properties.

Similar to the paediatric context, standard practices in managing adult primary wounds were attributed to convention and dogma. Professionals proposed a number of theoretical advantages and disadvantages of the default dressings they used, but acknowledged the lack of evidence in this area. The theories proposed were variable—particularly with respect to SSI prevention; while some suggested that dressings may prevent SSI by creating a barrier between the wound and sources of contamination, others suggested that dressings might promote the ideal conditions for bacteria to thrive:

Obstetric surgery nurse 2008: So from my experience I would say a wound dressing is to protect the wound, and basically it's to prevent infection, that's what I would believe it to do from my nursing practice and midwifery. The whole reason they have a dressing is to protect that area from, you know, foreign bodies and bacteria and the environment.

General surgeon 1005: I suppose the, the accepted dogma would be that that protects the wound from infection because it's sealed the skin…I don't think…I would be very surprised if it makes much of a difference in terms of infection rates cos I sometimes think dressings are sort of keep a wound warm and moist and that may actually provide ideal incubation kind of environment for bugs.

Professionals' views were more polarised towards dressings being beneficial with respect to practical considerations (eg, absorption of wound exudate), although some posited that exposed wounds may be practically easier to monitor and assess. Overall, all theories for/against dressing use on primary wounds were tempered with uncertainty and an acknowledged need for evidence:

General surgery nurse 3012: Unless we run trials to prove whether they [dressings] are effective or not we'll never know will we?

General surgeon 1004: I would be prepared to consider [not applying a dressing] in the context of a research trial, because I accept that the evidence base is poor and a lot of it reflects personal practice.

#### Acceptability of an RCT

All professionals supported the prospect of an RCT that included a ‘no dressing’ group, as long as this focused exclusively on primary surgical wounds. All accepted that there was clinical equipoise about the relationship between dressing use and SSI events in these wounds. Nonetheless, some expressed reticence about practical issues such as management of wound exudate (eg, blood, tissue fluid), and questioned whether patients would accept the possibility of not receiving a dressing:

Obstetric surgeon 2001: There can be you know, a bit of general ooze from the skin and fat layer which can be quite difficult to stop. So if you wanted to put a pressure dressing on you would need to put a dressing on…

General surgery nurse 1002: They [patients] might not like the idea of not having their wound covered […]. Very often they just don't like the look, or they worry about the wound opening up. Even though there's no clinical sign of the wound opening up, they get very concerned about their wounds.

Conversely, there were also suggestions that patients might welcome the opportunity of avoiding the potentially painful experience of dressing removal, and may begin the psychological recovery process sooner if confronted with the exposed wound from the outset:

Obstetric nurse 2009: I expect some of them [patients] will think “Oh good, I haven't got to peel it off in the shower,” because that's not very pleasant either.

General surgery nurse 1001: Even if it is just a very, very simple dressing they can look at that as a reason…(like) “Oh can I shower with that? Or, “Is that going to stop me from coughing?” So yes, it can be restrictive and detrimental psychologically to their recovery. I think not having dressings will improve that. I think it will improve the psychological recovery for patients.

Taken together, professionals' accounts suggested that there were balanced reasons for/against dressing use, although the trial team would need to carefully consider the inclusion/exclusion and withdrawal criteria in relation to wound exudate.

Most patients anticipated that they would participate in an RCT of different dressing strategies, citing altruistic reasons or an indifference about how their wound was managed. Many of these patients had undergone, or were scheduled to undergo, major surgery. To some, dressing considerations were perceived as trivial when considered in this hypothetical context. Some raised the possible practical consequences of foregoing a dressing (eg, wound-closure materials catching on clothing, absorption of exudate), though only a minority felt these concerns would deter them from RCT participation. Those who were unfavourable towards RCT participation tended to amalgamate practical considerations with psychological concern (or ‘worry’, as reported by patients):

General surgery participant 1032: I think I would, just because of the way that I…because of everything that I…you know like knocking, catching, be a bit worried maybe.

Obstetric surgery participant 2001: The day after, two days after, that's fine, I don't mind not having a dressing but straight after surgery, especially because it's leaking blood and all the rest of it, I would want it covered. It would worry me.

A few patients who had prior experience of receiving a dressing after surgery questioned whether foregoing a dressing would be detrimental to wound healing or increase the risk of infection. When it came to clinical considerations, however, acceptability of the RCT appeared to be entwined with whether their clinical team (whom they trusted) supported trial participation:

General surgery patient 1031: I (would) think “Oh they know best” and I just carry on with it and do what they want me to do.

### Defining potential comparison groups

Given that there was overall support for the RCT in principle, further research explored the scope and definition of ‘simple’, ‘complex’ or ‘no dressing’, and how these might work in the context of the proposed pilot RCT.

#### Interpretations of ‘dressing’ and ‘no dressing’

Interviews revealed variable definitions of what constituted a ‘dressed’ and ‘undressed’ wound. Professionals sought clarification on whether application of non-adherent products to absorb exudate (eg, gauze) would breach a ‘no dressing’ allocation, and queried how partially covered wounds should be categorised. This raised the possibility that patients in a pilot RCT may be treated inconsistently if allocated to a ‘no dressing’ group:

Paediatric surgeon 1013: I suppose I think of a dressing as something that's been used to deliberately cover up and, in a sense, cover up the wound…and with Steri-strips you leave gaps between them.

Obstetric registrar 2019: Vaginal operations there are no dressings even though often then you use pads. Is that a dressing because it is used to cover the wound? Would that be considered a dressing? I would say that's interesting: because you don't stick it on, it's not a dressing.

Initial interviews introduced the unexpected use of glue as a covering over primary wounds. Although the BNF currently lists glue as a wound-closure product, some professionals reported that this functioned as a ‘dressing’ when applied over an already closed wound. The dual use of glue as a wound-closure product and dressing was recognised as a potential source of confusion in a future pilot RCT:

General surgery nurse 1014: I would say dressings are something that occludes the skin, that covers the wound…Steri-strips don't. Glue doesn't, but can do, but doesn't necessarily…

#### Interpretations of ‘simple’ and ‘complex’ dressings

Interview informants encountered difficulties in interpreting the ‘simple’ and ‘complex’ dressing categories specified in the funder's call. Professionals did not use dressing classifications in routine management of primary wounds. The tendency to use a single default dressing in this context negated the need for any formal dressing classification in day-to-day practice:

General surgeon 1005: Hmm, does that mean anything to me? No, but as a term… not really, but at the same time simple dressing(s) probably are the ones we use every day…

Despite professionals' unfamiliarity with the proposed dressing classifications, most could intuitively envisage the types of dressings each group might encompass. ‘Simple’ was thought to be an apt descriptor for the default dressings used on primary wounds, which were consistently described as adhesive coverings that could be absorptive or non-absorptive. ‘Complex’ dressings were thought to have specialised functions that extended beyond wound coverage and passive absorption of exudate, although informants had limited experience of using these:

General surgery nurse 1001: You've basically got dressings that are simply a covering or a fixation that are literally just covering up something. They don't have any properties other than they are covering up something […]. Then you've got some dressings that are very specific—but we do occasionally use them—that have got antimicrobial properties, so they're trying to stop an infection. By far the one we use the most is the first, so just a simple covering. I would say 80 to 90% of our wounds were just simple coverings.

#### Developing pragmatic definitions

The inconsistent interpretation of the proposed wound dressing strategies, including the variable interpretations of ‘dressing’, reiterated the need for clear-cut definitions that could be used in the pilot RCT. Definitions needed to be comprehensible to front-line professionals and sufficiently exhaustive to encompass existing dressings mentioned in the BNF. Definitions were iteratively developed across three study management group meetings, supported by emerging data from interviews (table 4). Working definitions were scrutinised by considering the areas of controversy raised in relation to what constitutes a ‘dressing’ (eg, issues of adherence, extent of wound coverage), and professionals' intuitive interpretations of ‘simple’ and ‘complex’ dressings. Wound care experts reviewed the final pragmatic definitions during a presentation at a national meeting and a study steering committee meeting. Organising BNF-listed dressings into these pragmatic categories revealed that ‘simple’ dressings encompassed products that cost no more than £4 per item, while ‘complex’ dressings all cost over £4 per item. This aligned with details specified in the funder's commissioned call, which classed ‘simple’ dressings as ‘inexpensive’, and ‘complex’ dressings as ‘expensive’ (see online supplementary information 4).

**Table 4:** Bluebelle pragmatic definitions of dressing strategies

Dressing strategy	Bluebelle pragmatic definition
No dressing	The absence of any covering applied to a closed wound at the end of the operation. If there is exudate from the wound after surgery a simple gauze swab (basic wound contact dressing) may be applied/taped to the area of the wound that is oozing. *Note: Steristrips would fall under this category if they do not cover the entire wound*.
A simple dressing	A covering (opaque or transparent) directly applied over the entirety of an already closed wound at the end of the operation. It has adherent properties around its perimeter or its entire surface, and may have pads to absorb exudate. It will not be amorphous, have silicone, hydrocolloid or foam. *Note: Steristrips would fall under this category if they cover the entire wound*.
A complex dressing	A covering that is directly applied over the entirety of an already closed wound at the end of the operation which has intended advanced practical properties and/or therapeutic properties. This may include amorphous material, silicone, hydrocolloid, foam, antimicrobials and it will exclude topical negative pressure therapy.

10.1136/bmjopen-2016-012635.supp4 Supplementary data

### The relevance of comparison groups

#### Relevance of ‘simple’ and ‘complex’ dressings

Having developed a pragmatic categorisation of dressings, questions remained about whether the proposed dressings groups were relevant to current practice. Interviews revealed strong consensus that a future RCT should omit ‘complex’ dressings on the basis that these were not used in routine management of primary wounds. The survey was developed in response to this finding, and subsequently provided evidence that confirmed this (reported in full elsewhere).^8^ In summary, of the 1794 wounds surveyed, 1706 (96%) received a dressing, and 63 (4%) did not receive a dressing. In total, 1206/1706 (71%) of the dressed wounds were covered using a product that matched the pragmatic ‘simple dressing’ classification, with only 18 (1%) wounds dressed with a product matching the ‘complex’ dressing definition.

Further justification for omitting ‘complex’ dressings was associated with concerns about generalisability, given their heterogeneous nature:

Obstetric surgeon 2001: But once you start into complex dressings then there must be so many different kinds of complex dressings […]. Their manufacturers will still be able to turn round and say ah but this is irrelevant to our dressing, because ours is impregnated with Beetlejuice and therefore is much better than either that complex dressing, that simple dressing or no dressing at all, and that's the only thing really isn't it? So simple against none will be much cleaner and easier.

#### Glue as a dressing

Most professionals who were asked about glue acknowledged that it could function as a dressing, if applied over an already closed wound, but many did not intuitively associate glue with the term ‘dressing’. This had implications for the accuracy of reporting in the wound dressing survey. To address this difficulty, the survey included a separate item that asked respondents to state ‘any other products’ applied over the closed surgical wound. Glue was subsequently found to be used as a dressing in 485/1706 (28%) of the wounds sampled, accounting for most of the wounds that had not received a ‘simple’ dressing.^8^

In alignment with the survey findings, interviews revealed considerable variation in professionals' familiarity and experience of using glue as a dressing. This was the sole dressing product that was reportedly actively selected by some of the general surgeons interviewed. Most professionals working in general surgery were at least familiar with use of glue as a dressing, while some working in obstetrics found it hard to conceptualise glue as a dressing:

Obstetric consultant 2001: I have no understanding of glue. I know it is used in some contexts, particularly plastics, A&E, facial stuff, I think. I've never seen it used. I don't think anyone is using it in gynaecology or obstetrics in any hospital I've ever worked in in my thirty-year career […]. I've never heard of it being used as a dressing…I've thought about it with you here, and I uh, I don't quite get the concept.

There was widespread support for evaluating glue's effectiveness as a dressing. Professionals who used glue expressed a range of benefits, including ease of application, ease of movement for patients, and the ability to see the wound clearly. There was nonetheless an acknowledged need to assess its effectiveness in an RCT—particularly given its high cost in comparison to ‘simple’ dressings. Updating the Cochrane review to include ‘glue as a dressing’ revealed only two relevant RCTs,^12^ ^13^ with no conclusive evidence to show whether glue is beneficial over other dressing products, or leaving wounds exposed.^2^ This solidified the decision to include ‘glue-as-a-dressing’ in the forthcoming pilot RCT, in place of ‘complex dressings’. Glue will need to be applied over the entirety of an already closed wound to be classified as functioning as a dressing (ie, similar to the pragmatic definition of ‘simple dressing’).

### Outcome measures for the major RCT

As indicated earlier, patients and professionals noted that practical issues and patient satisfaction were key considerations for weighing out future evidence for and against dressing use. Two new outcome measure tools were therefore developed to capture the practical aspects of wound management, and patients' subjective experiences of the wound.^14^ These measures will be used to collect outcomes in the pilot RCT (phase B), alongside other specifically developed patient-centred tools to capture and measure SSI events,^15^ ^16^ in line with the original research brief.

### Final trial design

The final proposed RCT to be piloted in phase B will compare ‘simple’ dressings, ‘no dressing’ and ‘glue-as-a-dressing’ in primary abdominal surgical wounds, based on pragmatic definitions developed in phase A. In the event of wound exudate, the protocol will state that a simple gauze swab can be applied to the area that is oozing/bleeding. This swab may be taped in place, but not around its entire perimeter, and will not have therapeutic properties.

The main outcomes for the pilot RCT will include whether a patient is eligible, randomised and retained at 30 days post surgery. The pilot RCT will also seek to examine the feasibility of collecting complete and valid data for outcomes (eg, SSI rates) that will potentially be used in a subsequent large trial (as developed in phase A). Wounds will be examined by blinded assessors in the pilot and future main RCT. The pilot RCT will also include a nested methodological study to assess whether knowledge of dressing strategy allocation influences surgeons' approaches to wound closure. This will have implications for designing approaches to minimising performance bias in a future definitive RCT. Embedded qualitative research will investigate facilitators and barriers to recruitment and adherence to allocated dressing groups to further inform the feasibility of a large-scale RCT. A value of information analysis will be conducted alongside the pilot RCT to assess whether a full-scale RCT would be cost-effective from the perspective of the UK NHS.^17^

## Discussion

This mixed-methods study has demonstrated the benefits of incorporating preliminary feasibility work to optimise trial design, enhancing relevance to current practice. Novel insights into surgical wound dressing practices informed changes to an anticipated pilot RCT of wound dressing strategies. Dressing practices were found to be ingrained in surgical specialties, although clinical professionals acknowledged the need for evidence. Dressing selection for primary wounds was found to be a largely passive process, with most surgeons simply applying a ‘default’ dressing that was available in theatre. These default dressings were classified as ‘simple’. ‘Complex’ dressings were not used by any of the participating hospitals, and were interpreted as irrelevant to primary surgical wound care. Unexpectedly, glue did appear to be used as a dressing by some surgeons, despite the lack of evidence to support its use. Healthcare professionals and patients acknowledged the uncertainties around dressing use and SSI prevention, but reported that dressings may also have purely practical and/or psychological benefits or drawbacks.

Phase A of the Bluebelle study has highlighted gaps and contradictions in the research funder's topic brief against which the study was commissioned. The findings have altered the design of the pilot RCT that was originally planned, and informed aspects of the design that could not be specified at the outset. The pilot RCT originally intended to assess the feasibility of an RCT comparing ‘simple’, ‘complex’ and ‘no dressing’, with SSI as the primary outcome. Interview and survey findings indicated that the trial comparison groups needed to change in order for the pilot RCT to be pragmatic and relevant to current practice. The pilot RCT will now compare ‘simple dressings’, ‘glue-as-a-dressing’ and ‘no dressing’ on primary abdominal surgical wounds. Issues of practicality and acceptability were core considerations for patients and the surgical teams, thus prompting development of secondary outcomes to capture and measure these data.

A key strength of phase A was the mixed-methods approach, which allowed for triangulation of emerging findings and enabled a more rounded view of current practice. Qualitative methods provided depth of understanding and allowed for emergence of new lines of enquiry that might otherwise have gone unexplored. The approach to conducting the research and reporting findings in an iterative manner allowed the team to explore new avenues and verify emerging concepts and theories—both in a qualitative and quantitative manner. Sharing emerging findings among the multidisciplinary team iteratively allowed new objectives to be drawn up and allocated to the relevant methodological team, depending on the nature and scope of enquiry. This enabled the merits of working in a multidisciplinary team to be fully realised.

There were several limitations to this study. The qualitative findings—particularly exploration of trial acceptability—were limited by their reliance on hypothetical scenarios. Prior research has indicated that while surgeons may show support for research in principle, preferences or biases can lead to intentional or unwitting influences on recruitment and trial conduct.^18^ ^19^ Similarly, patients may have been inclined to produce socially desirable responses, and may react very differently if actually presented with the prospect of randomisation. The pilot RCT will therefore include embedded qualitative research to investigate challenges with recruitment and adherence to interventions as they occur in real time.^20^ Furthermore, the pragmatic definitions of dressing strategies were not developed through formal consensus methods, and have not yet been piloted. Application of the definitions in practice may call for further clarification and refinement. This will be assessed in the pilot RCT. Finally, while the insights emerging from this study would not have been possible without in-depth qualitative investigation, it should be noted that the findings are limited to UK secondary and tertiary care hospitals. It is also possible that clinicians' perspectives may differ on the basis of the volume and nature of procedures they predominantly conduct (eg, laparoscopic vs open surgery). The clinicians we interviewed routinely performed both types of operation, though it is possible that their activities (and thus experiences) were weighted towards a particular type of operation. If time and resources were not constrained, sampling would have taken place across a larger number of centres, to provide opportunities to build a more comprehensive sample of maximum variation, based on factors such as surgical subspecialty.

Given the plethora of dressing products on the market,^21^ it was imperative that the dressing comparison groups in the pilot RCT were reasoned, pragmatic and relevant. There is little empirical evidence detailing the commonality or breadth of dressing types used for surgical primary wounds, let alone the factors that influence choice of dressing. Published commentaries have alluded to dressing choice being driven by individual surgeon preference,^22^ ^23^ and clinical guidelines stipulate a number of factors professionals should consider in ‘dressing selection’.^23^ These sources suggest that dressing selection is reasoned, but a key finding from this study was the minimal ‘decision-making’ that often took place in dressing selection for primary wounds. While surgeon preference may not be as key an issue as anticipated, our findings raise the potential challenge of negotiating institutional barriers in the event that the proposed dressing types (eg, glue-as-a-dressing) are not available in RCT centres. The pilot RCT will provide an opportunity to identify solutions to these issues, should they be encountered.

Our findings have contributed original insights into the types of dressings currently used for surgical abdominal wounds, and the ways in which these are conceptualised by front-line professionals. The use of glue as a dressing was unexpected, though it is unclear if this product had been included in prior studies of dressing strategies, given the considerable variation in professionals' interpretations of what may constitute a ‘dressing’. None of the previous RCTs of dressing strategies has provided a clear definition of the intervention groups compared, raising questions around involvement of products such as glue, and the consistency of how wounds were treated in the trial comparison groups. Development of pragmatic definitions of ‘dressing’ and ‘no dressing’ in this feasibility study will inform a clear protocol for evaluating dressing strategies in the forthcoming pilot.

The qualitative research in particular was central to this study, informing and instigating other avenues for investigation. There is growing awareness of the value of embedding qualitative research in RCTs, though most published research to date has focused on issues of acceptance, adherence and patient experience as the trial proceeds.^24^ There are comparatively few published accounts of qualitative research at the pre-trial stage, whether in the lead up to pilot or main RCTs.^24^ The value of pre-trial qualitative research may be particularly heightened in studies that answer research funders' commissioned calls. As demonstrated by this study, the details of these calls cannot be taken for granted; investigators should consider scope for feasibility work that not only assesses whether a proposed RCT can be delivered, but considers the relevance of the research questions proposed. Further, the Bluebelle case study raises questions about the extent to which feasibility study protocols can be preplanned. New substudies in Bluebelle were initiated in response to emerging findings that could not have been anticipated. Permitting a degree of flexibility in feasibility study protocols may enhance their potential to fully address their aims and objectives, especially in studies with a strong exploratory focus.

This study highlights the value of conducting pre-trial research at the feasibility stage of RCTs, with respect to ensuring that the end product—the RCT—is maximally applicable, and answers the clinical and policy questions of greatest interest/priority. The feasibility research conducted in phase A has helped to optimise the design of an ambitious pilot RCT which challenges and questions an ingrained surgical practice. This was made possible by the qualitative methodology at the core of the study, which informed further questions and use of other research methodologies. The changes made to the pilot RCT will go some way to ensuring that a future RCT is relevant to front-line clinicians and patients. While there are still questions around feasibility, the pre-pilot work has addressed fundamental issues that may otherwise have prevented the study from informing the funder's decision about commissioning a large-scale RCT.
