# Correction: *Bluebelle study (phase A): a mixed-methods feasibility study to inform an RCT of surgical wound dressing strategies*

**DOI:** 10.1136/bmjopen-2016-012635corr1

**Published:** 2018-02-10

The Bluebelle Study Group, the Severn and Peninsula Audit and Research Collaborative for Surgeons, and the West Midlands Research Collaborative. Bluebelle study (phase A): a mixed-methods feasibility study to inform an RCT of surgical wound dressing strategies. *BMJ Open* 2016;6:e012635. doi: 10.1136/bmjopen-2016-012635

The study group for this research agreed a collaborative authorship policy, as stated in the title of the manuscript. The author byline should have read:

Rooshenas L, The Bluebelle Study Group, the Severn and Peninsula Audit and Research Collaborative for Surgeons, and the West Midlands Research Collaborative.

