# Supplementary material for: Bluebelle study (phase A): a mixed-methods feasibility study to inform an RCT of surgical wound dressing strategies
Source: BMJ Open. 2016 Sep 22;6(9):e012635. doi: 10.1136/bmjopen-2016-012635 (PMC5051448; doi:10.1136/bmjopen-2016-012635)
Supplement: Supplementary data [file bmjopen-2016-012635supp1.pdf]

HTA no 12/200

Dressings for the prevention of surgical site infection

Introduction

The aim of the HTA programme is to ensure that high quality research information on the effectiveness, costs and broader impact of health technology is produced in the most efficient way for those who use, manage, provide care in or develop policy for the NHS. Topics for research identified and prioritised to meet the needs of the NHS. Health technology assessment forms a substantial portfolio of work within the National Institute for Health Research and each year about fifty new studies are commissioned to help answer questions of direct importance to the NHS. The studies include both primary research and evidence synthesis.

Research Question:

What is the clinical and cost-effectiveness of dressings for the prevention of surgical site infection? When is a dressing necessary?

1. **Intervention:** Interactive dressings (applicants to define and justify).
2. **Patient Group:** Elective adult patients with non-infected clean surgical wounds healing by primary intention (e.g. abdominal/ vascular surgery/ caesarean section/ orthopaedic wounds). Researchers to define the term “non-infected”.
3. **Setting:** Secondary and community care.
4. **Control:** Simple dressings (for instance simple gauze or polyurethane adhesives without any antiseptic). Third arm with no dressing. Standard infection control practices should be applied to all participants.
5. **Study design:** A feasibility study to develop the parameters of a subsequent two or three-armed trial. The two armed study would be simple dressings versus complex dressings. The feasibility study should find out if there is equipoise for a third “no dressing” arm; and test the ability to randomise. The feasibility study should also develop other aspects of the trial design such as the eligibility criteria and the outcome measures.
6. **Important outcomes:** The suitable study population; ability to recruit; identification of priority outcomes for clinicians and patients; feasibility of randomising; costs. An outline design for a subsequent trial should be developed but any such trial will be commissioned separately by the programme.
7. **Minimum duration of follow up:** 1 month.

Background information for potential applicants:

There are many dressings available, and there is rapid pace of change in this field. However, current studies are not adequate to show convincing differences. This study seeks evidence about whether complex and costly dressings are more cost-effective than simple and inexpensive alternatives, or no dressings. Previous trials have produced no significant results to indicate a difference in benefit.

Therefore this research is important to show in which circumstances these complex/expensive dressings are necessary; rather than the current practice where these dressing types are being overused at significant expense to the NHS.

Notes to Applicants

The NIHR Health Technology Assessment programme is funded by the NIHR, with contributions from the CSO in Scotland, NISCHR in Wales, and the Public Health Agency in Northern Ireland. Researchers from Northern Ireland and Scotland for certain NICE related calls should contact NETSCC to discuss their eligibility to apply.

For many of the questions posed by the HTA Programme, a randomised controlled trial is likely to be the most appropriate method of providing an answer. However, there may be practical or ethical reasons why this might not be possible. Applicants proposing other research methods are invited to justify these choices.

Applicants are asked to:

1. Follow the Medical Research Council's Good Clinical Practice guidelines (http://www.mrc.ac.uk/Utilities/Documentrecord/index.htm?d=MRC002416) when planning how studies, particularly RCTs, will be supervised. Further advice specific to each topic will be given by the HTA Programme at full proposal and contract stages.

2. Note that trials involving medicinal products must comply with "The Medicines for Human Use (Clinical Trials) Regulations 2004". In the case of such trials, the DH expects the employing institution of the chief investigator to be nominated as the sponsor. Other institutions may wish to take on this responsibility or agree co-sponsorship with the employing institution. The DH is prepared to accept the nomination of multiple sponsors. Applicants who are asked to submit a full proposal will need to obtain confirmation of a sponsor(s) to complete their application. The DH reserve the right to withdraw from funding the project if they are not satisfied with the arrangements put in place to conduct the trial.

The MHRA (info@mhra.gsi.gov.uk, http://www.mhra.gov.uk) can provide guidance as to whether your trial would be covered by the regulations. The DH/MRC website (http://www.ct-toolkit.ac.uk/) also contains the latest information about Clinical Trials regulations and a helpful FAQ page.

In line with the government's transparency agenda, any contract resulting from this tender may be published in its entirety to the general public. Further information on the transparency agenda is at: http://transparency.number10.gov.uk/#

Applicants are recommended to seek advice from suitable methodological support services, at an appropriate stage in the development of their research idea and application. It is advisable to make contact at an early a stage as possible to allow sufficient time for discussion and a considered response.

The NIHR Research Design Service (http://www.nihr.ac.uk/infrastructure/Pages/infrastructure_research_design_services.aspx) can advise on appropriate NIHR programme choice, and developing and designing high quality research grant applications.

Clinical Trials Units are regarded as an important component of any trial application and can advise and participate throughout the process from initial idea development through to project delivery and reporting. NETSCC CTU Support Funding (http://www.netscc.ac.uk/supporting_research/CTUs) provides information on the units receiving funding from the NIHR to collaborate on research applications to NIHR programmes and funded projects. In addition UKCRC CTU (http://www.ukcrc-ctu.org.uk) provides information and searchable information resource on all registered units in the UK.

Research networks

The HTA Programme expects, where appropriate, that applicants will work with the relevant research network.

Making an application

If you wish to submit an outline proposal on this topic, complete the on-line application form at http://www.hta.ac.uk/funding/standardcalls/index.shtml and submit it on line by **18th December 2012**. Applications will be considered by the HTA Commissioning Board at its meeting in **March 2013**. For outline applications, if shortlisted, investigators will be given a minimum of eight weeks to submit a full proposal.

Applications received electronically after <u>1300 hours</u> on the due date will not be considered.

Please see GUIDANCE ON APPLICATIONS overleaf.

Guidance on applications

Required expertise

HTA is a multidisciplinary enterprise. It needs to draw on the expertise and knowledge of clinicians and of those trained in health service research methodologies such as health economics, medical statistics, study design and qualitative approaches. The HTA programme expects teams proposing randomised controlled trials to include input from an accredited clinical trials unit, or one with equivalent experience. Applicants are also expected to engage a qualified Trial Manager for appropriate projects. A commitment to team working must be shown and applicants may wish to consider a collaborative approach between several institutions.

Public involvement in research

The HTA programme recognises the benefit of increasing active involvement of members of the public in research and would like to support research projects appropriately. The HTA programme encourages applicants to consider *how* the scientific quality, feasibility or practicality of their proposal *could* be improved by involving members of the public. Examples of how this has been done for health technology assessment projects can be found at http://www.hta.ac.uk/PPIguidance/. Research teams wishing to involve members of the public should include in their application: the aims of active involvement in this project; a description of the members of the public (to be) involved; a description of the methods of involvement; and an appropriate budget. Applications that involve members of the public will not, for that reason alone, be favoured over proposals that do not but it is hoped that the involvement of members of the public will improve the quality of the application.

Outcomes

Wherever possible, the results of HTA should provide information about the effectiveness and cost-effectiveness of care provided in its usual clinical setting and for the diverse subjects who would be eligible for the interventions under study. The endpoints of interest will in most cases include disease specific measures, health related quality of life and costs (directly and indirectly related to patient management). Wherever possible, these measurements should be made by individuals who are unaware of the treatment allocation of the subjects they are assessing. We encourage applicants to involve users of health care in the preparation of their proposal, for instance in selecting patient-oriented outcomes. Where established Core Outcomes exist they should be included amongst the list of outcomes unless there is good reason to do otherwise. Please see The COMET Initiative website at www.comet-initiative.org to identify whether Core Outcomes have been established. A period of follow up should be undertaken which is sufficient to ensure that a wider range of effects are identified other than those which are evident immediately after treatment. These factors should guide applicants in their choice of subjects, settings and measurements made.

Sample size

A formal estimate should be made of the number of subjects required to show important differences in the chosen primary outcome measure. Justification of this estimate will be expected in the application.

Communication

Communication of the results of research to decision makers in the NHS is central to the HTA Programme. Successful applicants will be required to submit a single final report for publication by the HTA programme. They are also required to seek peer-reviewed publication of their results elsewhere and may also be asked to support NETSCC, HTA in further efforts to ensure that results are readily available to all relevant parties in the NHS. Where findings demonstrate continuing uncertainty, these should be highlighted as areas for further research.

Timescale

There are no fixed limits on the duration of projects or funding and proposals should be tailored to fully address the problem (including long-term follow-up if necessary). Applicants should consider however that there is a pressing need within the NHS for this research, and so the duration of the research needs to be timely.

Feasibility and Pilot studies

We expect that when pilot or feasibility studies are proposed by applicants, or specified in commissioning briefs, a clear route to the substantive study will be described. This applies whether the brief or proposal describes just the preliminary study or both together. Whether preliminary and main studies are funded together or separately may be decided on practical grounds.

Feasibility Studies are pieces of research done before a main study. They are used to estimate important parameters that are needed to design the main study. Feasibility studies for randomised controlled trials may not themselves be randomised. Crucially, feasibility studies do not evaluate the outcome of interest; that is left to the main study. If a feasibility study is a small randomised controlled trial, it need not have a primary outcome and the usual sort of power calculation is not normally undertaken. Instead the sample size should be adequate to estimate the critical parameters (e.g. recruitment rate) to the necessary degree of precision.

Pilot studies are a version of the main study that is run in miniature to test whether the components of the main study can all work together. It is focused on the processes of the main study, for example to ensure recruitment, randomisation, treatment, and follow-up assessments all run smoothly. It will therefore resemble the main study in many respects. In some cases this will be the first phase of the substantive study and data from the pilot phase may contribute to the final analysis; this can be referred to as an internal pilot. Or at the end of the pilot study the data may be analysed and set aside, a so-called external pilot.

For a full definition of the terms 'feasibility study' and 'pilot study' visit the NETSCC website glossary page http://www.netscc.ac.uk/glossary/

Diagnostics and Imaging

In evaluating diagnostic and imaging techniques, the emphasis of the HTA programme is to assess the effect on patient management and outcomes (particularly where changes in management can be shown to have patient benefits). Improvements in diagnostic accuracy, whilst relevant, are not the primary interest of this commissioned research programme. Applicants should justify where they consider improvements in diagnostic accuracy to be relevant to these objectives. Where there is poor evidence to link diagnostic improvements to patient benefits, part of the primary research may be to assess the effects of such changes on patient outcome.

An assessment should also be made of changes in other resources (particularly other subsequent therapies) used as a result of changes in diagnostic methods.
