# Supplementary material for: Bluebelle study (phase A): a mixed-methods feasibility study to inform an RCT of surgical wound dressing strategies
Source: BMJ Open. 2016 Sep 22;6(9):e012635. doi: 10.1136/bmjopen-2016-012635 (PMC5051448; doi:10.1136/bmjopen-2016-012635)
Supplement: Supplementary data [file bmjopen-2016-012635supp2.pdf]

Biographies of researchers conducting qualitative data collection and analyses

Leila Rooshenas (LR), Christel McMullen (CM), and Daisy Elliott (DE) are female health services researchers, all of whom have a PhD in health services research, and at least 3 (WTE) years of post-doctoral qualitative research experience. All were post-doctoral research associates or fellows at the time of study conduct.

Jonathan Mathers (JM) is a male health services researcher, with a PhD in health services research, and at least 5 (WTE) years of post-doctoral qualitative research experience. JM was a senior lecturer at the time of study conduct. JM has broad based expertise in the application of qualitative and mixed methods research approaches for applied health and policy research. He is currently leading the qualitative research components for a number of clinical trial feasibility studies funded by the NIHR Health Technology Assessment and Research for Patient Benefit programmes.

All researchers involved in qualitative data collection/analysis approached this project as naive researchers, with little knowledge of the field or literature. We have no reason to believe that any of the researchers' personal experiences or life histories would have influenced their approaches to data collection and analysis in a noteworthy way.

Contact with study participants

LR, CM, DE, and JM had no prior contact or relationships with the majority of research participants prior to initiation of OPTIMA prelim. There were three exceptions, where all were familiar with three of the clinical professional interview informants who were members of the study trial management group. LR, CM, DE, and JM spoke with or met a number of the research participants in advance of interviews, but these encounters were limited to introductions.

Research participants all received study information sheets in advance of data collection processes, which explained why the research was being undertaken. Information sheets included no personal details about the researchers or their personal aspirations. As such, interview participants will not have been informed of any personal information about the researchers prior to being interviewed, although they will have known the identity of the researchers (through email correspondence), and thus had opportunity to read about researchers' research interests in advance (e.g. via the internet). Although this was not explicit in interviews, there is a possibility that participants' prior knowledge of researchers' interests influenced their accounts and responses.
