# Supplementary material for: Bluebelle study (phase A): a mixed-methods feasibility study to inform an RCT of surgical wound dressing strategies
Source: BMJ Open. 2016 Sep 22;6(9):e012635. doi: 10.1136/bmjopen-2016-012635 (PMC5051448; doi:10.1136/bmjopen-2016-012635)
Supplement: Supplementary data [file bmjopen-2016-012635supp3.pdf]

Supplementary Information 3- original and final topic guides for interviews

a) Original topic guides

INTERVIEW TOPIC GUIDE (CLINICAL STAFF)

Opening

Interviewer will re-iterate study information, answer any questions, and take written consent.

Background, interviewee details and ice breaker

e.g. details of interviewee's current position and role, working history in clinical role, experience etc.

Range / type of surgical procedures that the interviewee undertakes (surgeons) or cares for patients following (nurses)

Wound dressing use

Type of wound dressings that are currently used following these procedures.

Factors that affect choice of wound dressing e.g. patient, clinical and procedure-related.

The aims and expected outcomes of dressing use.

Perspectives on the clinical and cost-effectiveness of dressings.

Likely acceptability of a trial of dressing type

Including;

- Range of procedures that would be acceptable for inclusion
- Dressing comparisons that would be acceptable (simple vs complex, or no dressing)
- Perceived barriers to the successful conduct of a pilot and/or pragmatic RCT of dressing use, and specifically one with a 'no dressing' arm.

Perspectives on important outcomes to include in a trial of dressing type.

Closing

Interviewer checks understanding of any outstanding points, answers further questions, and checks to see if interviewee would like to receive a summary of findings.

INTERVIEW TOPIC GUIDE (PATIENTS)

Opening

Interviewer will re-iterate study information, answer any questions, and take written consent.

Background, interviewee details and ice breaker

e.g. relevant interviewee background and details of procedure that the interviewee has had / is scheduled to have (type of procedure, when, where, length of stay in hospital, recovery period).

Discuss the patient's expectations or experience of wound care and dressing use, including the use of and need for wound dressings.

Patient perspectives on a trial of dressing type

Explore the patient's attitudes toward a trial of dressing use including;

- whether patients like them would be likely to participate?
- what might influence choices to participate?
- how patients would feel about random allocation to dressing type, and specifically the possibility of receiving no wound dressing?
- Perceived barriers to a trial of dressing type.
- Patient perspectives on important outcomes to include in a trial of dressing use.

Closing

Interviewer checks understanding of any outstanding points, answers further questions, and checks to see if interviewee would like to receive a summary of findings.

b) Final topic guides

INTERVIEW TOPIC GUIDE- Perspectives on wound dressing (Clinical Staff)

Opening

Interviewer will re-iterate study information, answer any questions, and take written consent.

Background, interviewee details and ice breaker

- Details of interviewee's current position and role, working history in clinical role, experience etc.

Details of clinical speciality

- Explore the range /type(s) of surgical procedures that the interviewee undertakes (surgeons) or procedures for caring for patients following surgery (nurses/midwives).

Wound dressing use

Explore:

- Whether wound dressings are generally used in [speciality]? Any exceptions (e.g. specific procedures or scenarios)? *(Probe: how do their practices compare with peers? Comparisons within/across centres?).*
- What do you understand from the term 'dressing'? *[Explore perceptions of whether 'glue' and Steri-strips constitute 'dressings']*
- Why dressings are used/not used in [speciality/exceptions]? (*Probe: what are they supposed to achieve; are they effective in doing do? Cost effective?*)
- The type of wound dressings that are currently used following surgical procedures (where wound dressings are relevant). How would one classify these dressings? (*Probe: Simple? Complex? Definitions of simple and complex? Are there different sub-types of simple and complex dressings?)*
- Factors that affect choice of wound dressing (*Probe: patient, clinical, procedure-related, institutional and financial factors*).
- Process of wound dressing and follow up (*Probe: Who? Where? When?).*
- How informant feels about the prospect of 'no dressing' in procedures where these are currently used. Any potential impact on practice? (*Probe: closing wounds; follow-up, etc*.)

<u>Likely acceptability of a trial of dressing type</u>

Explore attitudes towards a trial of dressing use, including:

- Range of procedures that would be interesting/acceptable for inclusion; any procedures to avoid.
- Dressing comparisons that would be acceptable (simple, complex, no dressing). (*Note: ensure views on 'no dressing' option are thoroughly explored.)*
- Perceived barriers to the successful conduct of a pilot and/or pragmatic RCT of dressing use, specifically one with a 'no dressing' arm.
- Perspectives on important outcomes to include in a trial of dressing type.

<u>Closing</u>

Interviewer checks understanding of any outstanding points, answers further questions, and checks to see if interviewee would like to receive a summary of findings.

<u>**INTERVIEW TOPIC GUIDE- Perspectives on wound dressing (Patients)**</u>

<u>Opening</u>

Interviewer will re-iterate study information, answer any questions, and take written consent.

<u>Background, interviewee details and ice breaker</u>

- Interviewee background and details of procedure that the interviewee has had / is scheduled to have (type of procedure, when, where, length of stay in hospital, recovery period).

Expectations/experiences of wound care

Discuss the patient's expectations or experience of wound care and dressing use, including the use of and need for wound dressings. Explore:

- Expectations of wound management- dressing/no dressing/type of dressing? (*Probe: what are/were these expectations based on?)*
    - *Had you given any thought about the wound after surgery?*
    - *Did you have any expectations about whether or not you would have a dressing? If so, where do you think these expectations came from?*
- *[For patients who have received surgery]* Experiences of having a dressing/not having a dressing (*Probe: perceived influence on recovery and symptoms; practical considerations, etc.). Why do you think you had/did not have a dressing?*
- Thoughts/reactions to **alternative** wound management methods (i.e. dressing or no dressing in relation to what patient has experienced/what patient expects). (*Explore patient thoughts on impact this may have on previously mentioned issues (e.g. recovery, symptoms, practicalities*).

Patient perspectives on a trial of dressing type

[Explain: we are not sure whether it is better to apply a dressing over the wound after surgery, or leave it exposed to air]

Explore the patient's attitudes toward a trial of dressing use, including:

- Whether patients like them would be likely to participate? *(Reasons why/why not? Any reservations?)*
    - *Can you imagine your family and/or friends would participate in a study like this? (explore reasons)*
- What might influence choices to participate?
    - *Do you think you would have any questions about the study?*
    - *What kind of things might you want to know about in advance?*
- How patients would feel about random allocation to dressing type, specifically the possibility of receiving no wound dressing? *[Explain randomisation to patients, then ask…]*
    - *If you were in a group that didn't receive a dressing, do you think this would have any impact on what you do/how you behave after surgery?*
- Perceived barriers to a trial of dressing type. *How do you think we might get around this?*
- Perspectives on important outcomes to include in a trial of dressing use. *[Explain: We would like this study to help us answer the question of whether dressings should be used in patients such as yourself (and if so, what type of dressing is best). What do you think are the important factors we should consider when making any future recommendations on dressing use?]*
