# Supplementary material for: Bluebelle study (phase A): a mixed-methods feasibility study to inform an RCT of surgical wound dressing strategies
Source: BMJ Open. 2016 Sep 22;6(9):e012635. doi: 10.1136/bmjopen-2016-012635 (PMC5051448; doi:10.1136/bmjopen-2016-012635)
Supplement: Supplementary data [file bmjopen-2016-012635supp4.pdf]

Supplementary Information 4- Terminology and descriptions of dressings in BNF mapped onto Bluebelle pragmatic dressing categories

BNF term	BNF description	Bluebelle Study pragmatic dressing categories
**No dressing**	Not defined	**'No dressing'**<br>The absence of any covering applied to a closed wound at the end of the operation. If there is exudate from the wound after surgery a simple gauze swab (basic wound contact dressing) may be applied/taped to the area of the wound that is oozing.
**Basic wound contact**<br>i Low adherence<br><br>ii Simple absorbent	Low adherent absorbent dressings which are placed directly in contact with the wound. Can be either non-medicated or medicated.<br>Low adherent usually cotton pads which are placed directly in contact with the wound. Can be either non-medicated or medicated	**'A simple dressing'**<br>A covering (opaque or transparent) directly applied over the entirety of an already closed wound at the end of the operation. It has adherent properties around its perimeter, or, its entire surface and may have pads to absorb exudate. It will not be amorphous, have silicone, hydrocolloid or foam.

**Advanced**<br>i. Hydrogel<br>ii. Vapour permeable<br>iii. Soft polymer<br>iv. Hydrocolloid<br>v. Foam<br>vi. Alginate<br>vii. Capillary action<br>viii. Odour absorbent	An amorphous, cohesive topical application. Can be the shape of a wound<br>Allows the passage of water vapour & oxygen & but impermeable to water A soft silicone polymer, may/may not be adherent<br>A hydrocolloid layer on a vapour-permeable film/foam pad, adherent or not Dressings containing foam with or without plastic film-backing Dressings made from calcium alginate, or calcium sodium alginate. Hydrophilic fibres between two low-adherent wound-contact layers<br>Containing activated charcoal are used to absorb odour from wounds	**'A complex/advanced dressing'**<br>A covering that is directly applied over the entirety of an already closed wound at the end of the operation which has intended advanced practical properties and/or therapeutic properties. This may include amorphous material, silicone, hydrocolloid, foam, anti-microbials and it will exclude topical negative pressure therapy.
**Anti-microbial**	A dressing impregnated with antimicrobial agents	
**Specialised**	This includes protease modulating dressings for chronic wounds and silicone gel to be used to prevent hypertrophic scarring.	

**Complex adjunct**	Topical negative pressure (or vacuum-assisted) therapy	
**“Glue-as-a-dressing”**	Not described as a dressing	**“Glue-as-a dressing”**<br>Tissue-adhesive applied to an already closed wound at the end of the operation with no further dressings applied.
